# Supplementary material for: Splice-Junction-Based Mapping of Alternative Isoforms in the Human Proteome
Source: Cell Rep. Author manuscript; Available in PMC 2020 Jan 15. (PMC6961840; doi:10.1016/j.celrep.2019.11.026)
Supplement: 2 [file NIHMS1546469-supplement-2.zip › DF1/PXD010154/Liver/Liver_44_TSTA3_QEDDRRAEQIHNGPPHNSNFGYSYAK.pdf]

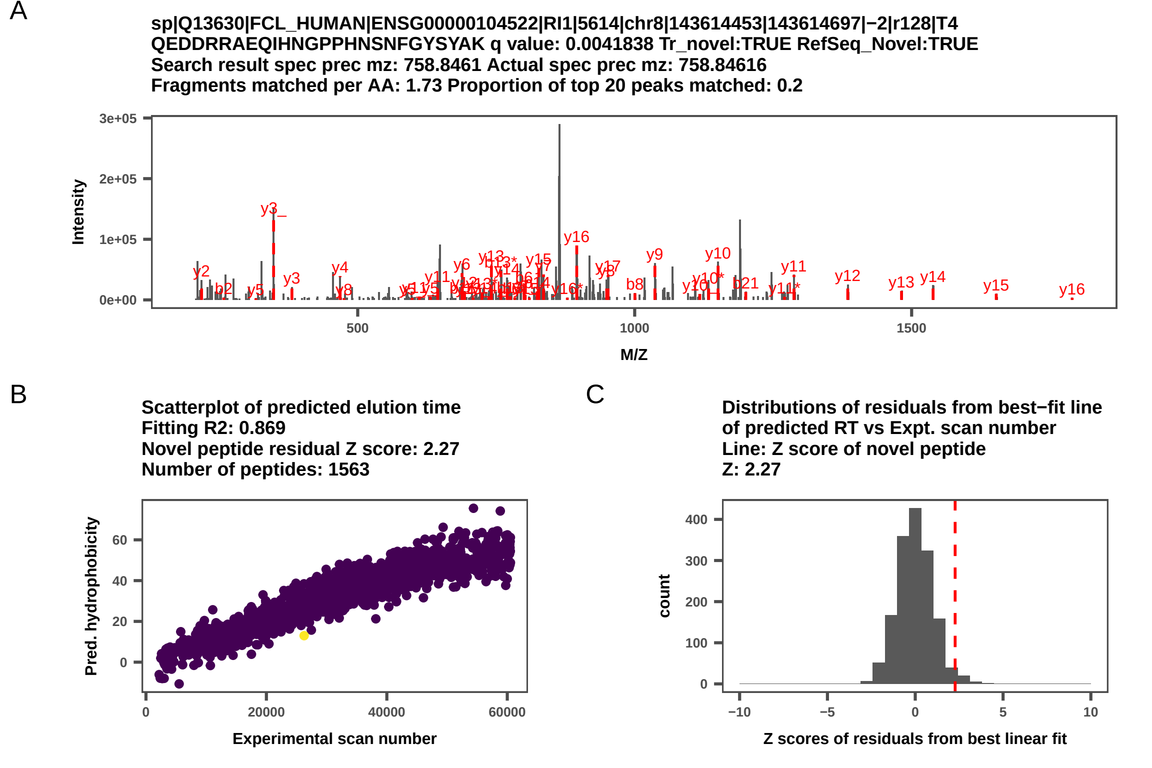
A
sp|Q13630|FCL_HUMAN|ENSG00000104522|RI1|5614|chr8|143614453|143614697|−2|r128|T4
QEDDRRAEQIHNGPPHNSNFGYSYAK q value: 0.0041838 Tr_novel:TRUE RefSeq_Novel:TRUE
Search result spec prec mz: 758.8461 Actual spec prec mz: 758.84616
Fragments matched per AA: 1.73 Proportion of top 20 peaks matched: 0.2
Intensity
3e+05
2e+05
1e+05
0e+00
M/Z
500
1000
1500
y3_
y16
y2
b2
y5
y3
y4
y8
y6
y11
y9
y10
b8
b21
y11
y12
y13
y14
y15
y16
B
Scatterplot of predicted elution time
Fitting R2: 0.869
Novel peptide residual Z score: 2.27
Number of peptides: 1563
Pred. hydrophobicity
60
40
20
0
Experimental scan number
0
20000
40000
60000
C
Distributions of residuals from best−fit line
of predicted RT vs Expt. scan number
Line: Z score of novel peptide
Z: 2.27
count
400
300
200
100
0
Z scores of residuals from best linear fit
−10
−5
0
5
10
